# Low Back Pain Caused by Traction Peripheral Neuropathy Due to Chronic Ankle Instability: Three Clinical Cases

**DOI:** 10.7759/cureus.65405

**Published:** 2024-07-26

**Authors:** Yusuke Hagiwara, Yumiko Natsume, Tomomi Wagatsuma, Tetsuya Hasegawa, Ryu Yoshida

**Affiliations:** 1 Orthopaedic Surgery, Toho Kamagaya Hospital, Chiba, JPN; 2 Orthopaedic Surgery, Oshima Medical Association Hospital, Kagoshima, JPN; 3 Research into Artifacts, Center for Engineering (RACE) School of Engineering, The University of Tokyo, Tokyo, JPN; 4 Orthopaedic Surgery, Cedars-Sinai Medical Center, Los Angeles, USA

**Keywords:** traction neuropathy, superficial peroneal nerve, straight leg raise test, ankle-foot orthoses, nerve block, retrograde neuropathic pain, ankle sprain, chronic ankle instability, non-specific low back pain, low back pain

## Abstract

Non-specific low back pain without identifiable causes on imaging is a common and frustrating problem for both patients and physicians. While proximal symptoms such as shoulder pain from distal upper extremity neuropathies such as carpal tunnel syndrome are well-known, peripheral neuropathy of the foot or ankle is rarely considered in the differential diagnosis for low back pain. This study aims to highlight the potential link between chronic ankle instability (CAI) and low back pain. We present three cases: a 32-year-old woman with chronic low back pain for over 10 years, a 59-year-old woman with transient low back pain after long drives, and a 42-year-old woman with acute low back pain while gardening. All patients had normal imaging studies but exhibited CAI on examination. Diagnostic modalities included the ankle anterior drawer test, application of ankle brace, superficial peroneal nerve (SPN) blocks, and assessment of the active straight leg raise (aSLR) angle. In the first case, low back pain disappeared after SPN neurolysis and ankle ligament reconstruction. The second case showed significant improvement in aSLR and pain reduction with SPN block and ankle brace. The third case experienced substantial pain relief with the use of an ankle brace. These findings suggest that addressing ankle instability and associated traction neuropathy can significantly alleviate low back pain symptoms. CAI may be an underrecognized cause of non-specific low back pain. Interventions such as ankle brace, SPN blocks, SPN decompression, and ankle ligament reconstruction can be effective for diagnosis and treatment, potentially offering relief for patients with chronic low back pain.

## Introduction

Non-specific low back pain, for which no specific causes can be identified via examination, is a frustrating problem for both patients and physicians. Upper limb peripheral neuropathy has long been known to manifest proximal symptoms [1]. For example, carpal tunnel syndrome can cause shoulder or neck pain [2-4]. There have also been reports of double crush syndrome [5], in which symptoms become marked when cervical spondylosis and carpal tunnel syndrome coexist, and, more recently, parallel crush syndrome (PCS) [3], which is caused by the coexistence of carpal tunnel syndrome and cubital tunnel syndrome. On the other hand, peripheral neuropathy at the foot or ankle is usually not included in the differential diagnosis for low back pain. We report three cases of non-specific low back pain caused by traction neuropathy due to chronic ankle instability (CAI).

## Case presentation

Clinical case 1

**Figure 1:**
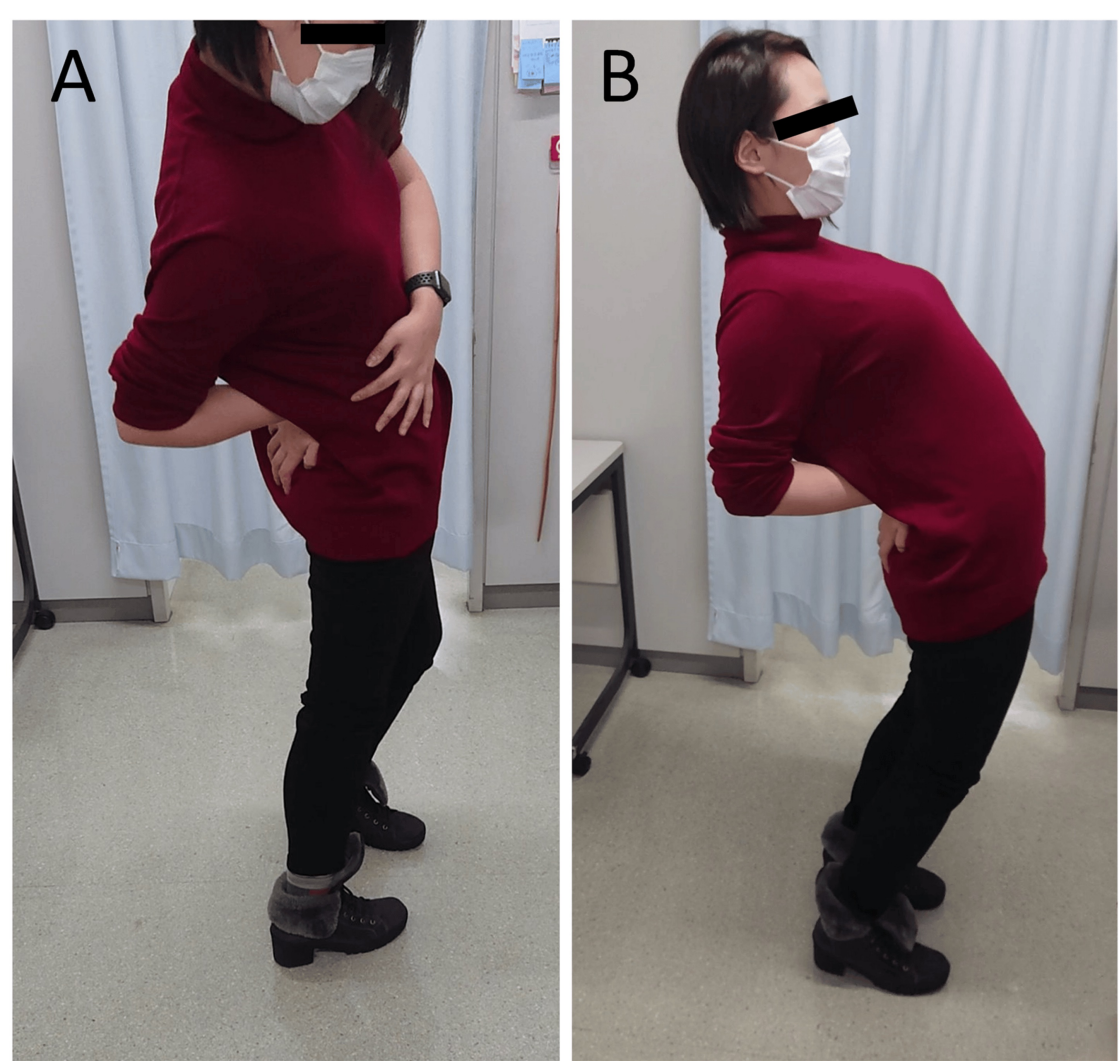
Improved lumber spine extension after superficial peroneal nerve treatment. (A) Right buttock pain limiting her back extension. (B) Improvement in lumbar extension after superficial peroneal nerve block.

A 32-year-old woman had chronic low back pain for more than 10 years, but radiography and magnetic resonance imaging (MRI) at another hospital had shown no abnormalities. She was referred to us with three weeks of right buttock pain worse with spine dorsiflexion. There were no contributing factors such as handling heavy objects or twisting. Examination revealed CAI (positive ankle anterior drawer test [6]) and tenderness in the lateral distal lower leg area, where the superficial peroneal nerve (SPN) exited the deep fascia. The pain during spine dorsiflexion disappeared temporarily after the application of an ankle-foot orthosis (Everstep 3 Ankle Support®, NIPPON SIGMAX CO., Tokyo, Japan) and SPN block (Figure 1) (Videos 1, 2).

**Video 1:** Echo-guided superficial peroneal nerve block.

**Video 2:** Improved lumber spine extension after ankle-foot orthosis and superficial peroneal nerve treatment.

**Figure 2:**
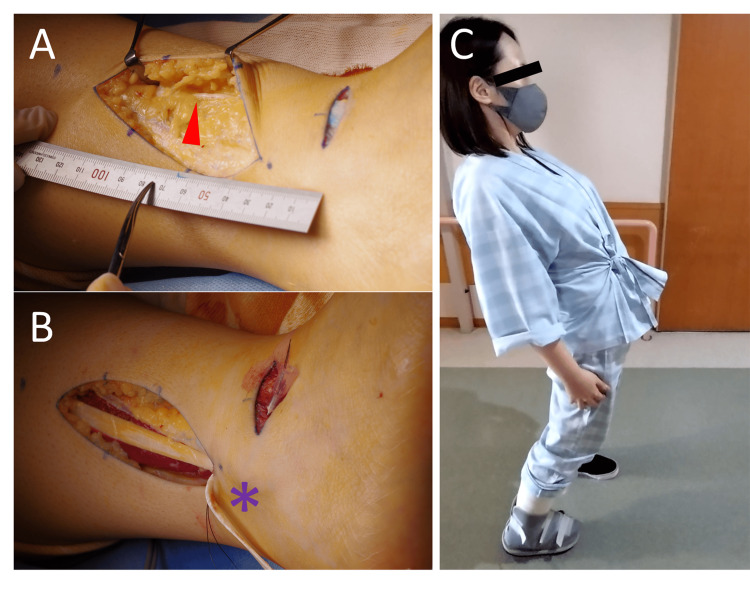
Intraoperative photos and improved lumbar spine extension after surgery. (A) SPN came out of the deep fascia 7.5 cm proximal to the lateral malleolus (▲). (B) The anterior talofibular ligament was reconstructed with half of the peroneus brevis tendon (*). (C) Improved lumbar spine extension two weeks after surgery.

At the second visit, the posterior tibial nerve (PTN) block at the tarsal tunnel also resolved low back pain, but the pain at this site was also caused by anterior-posterior instability of the ankle joint. Therefore, ankle ligament reconstruction with the Watson-Jones technique [7] and SPN neurolysis on the lateral side were performed, and low back pain disappeared in the early postoperative period (Figure 2).

**Figure 3:**
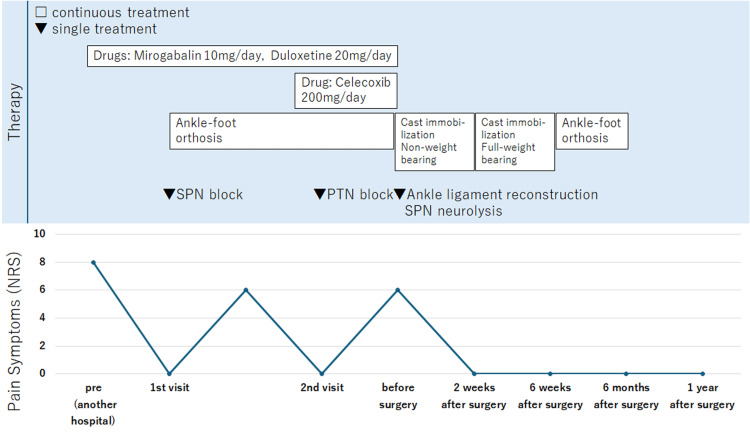
Graphical presentation illustrating the improvement in Case 1. SPN = superficial peroneal nerve

The dorsiflexion strength of the hallux and the angle of active straight leg raise (aSLR) also increased. One year postoperatively, there has been no recurrence of back or buttock pain (Figure 3).

Clinical case 2

A 59-year-old woman had repeatedly experienced transient low back pain after a long-distance drive. Although imaging studies at another hospital had shown no abnormalities, mild joint laxity had been noted. She presented to us with one week of persistent pain in the back as well as lateral thighs while walking. Examination revealed CAI on both sides. The symptoms were partially improved by wearing ankle-foot orthoses (Everstep 3 Ankle Support®, NIPPON SIGMAX CO., Tokyo, Japan) on both feet. Post-SPN block, the aSLR angle greatly improved from 20° to 45°, and low back pain while walking improved from 8/10 to 3/10 on a numerical rating scale (NRS) (Video 3).

**Video 3:** Improved active active straight leg raise test after superficial peroneal nerve treatment.

**Figure 4:**
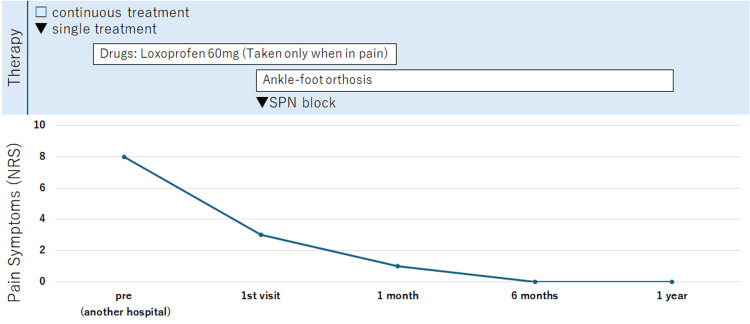
Graphical presentation illustrating the improvement in Case 2. SPN = superficial peroneal nerve

With ankle-foot orthoses, there was no recurrence of low back pain on long-distance driving for more than one year without additional nerve blocks (Figure 4).

Clinical case 3

**Figure 5:**
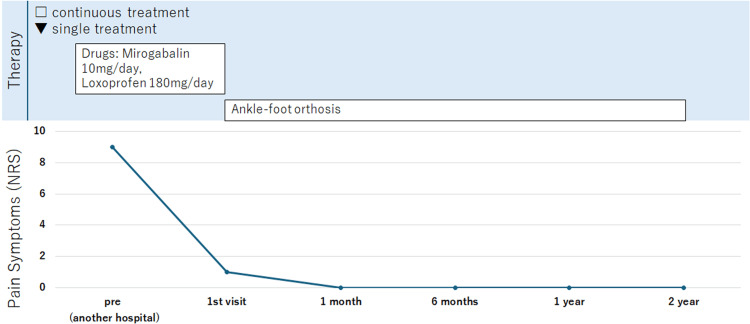
Graphical presentation illustrating the improvement in Case 3.

A 42-year-old woman experienced acute low back pain that occurred while gardening. Numbness was noted in the left lower limb, but an MRI at another hospital showed no abnormalities. Her symptoms worsened over the next month despite taking medications and a leave of absence for a hospital assistant, so she presented to us. The examination showed left CAI. With ankle-foot orthosis (Everstep 1 Ankle Support®, NIPPON SIGMAX CO., Tokyo, Japan), low back pain during getting up greatly improved to an NRS score of 1/10. Pain recurred once the orthosis was removed. With an ankle-foot orthosis, she has returned to her current work for more than two years without a recurrence of low back pain (Figure 5).

## Discussion

In all three of our patients, who repeatedly experienced low back pain recurrence, no abnormalities were detected via lumbar spine radiography or MRI, and the causes were unknown. In Case 1, both the tarsal tunnel and SPN blocks temporarily resolved the pain. Symptoms resolved with reconstruction of the lateral ligament of the ankle joint and SPN neurolysis. In Cases 2 and 3, pain was relieved by combined SPN block and ankle-foot orthoses and by an ankle-foot orthosis alone, respectively.

Non-specific low back pain, where workups including MRI do not demonstrate a cause, is unfortunately common [8]. In the absence of a clearly identifiable cause, it is typically treated with symptomatic treatment, such as medications, physical therapy, and lifestyle modifications.

As specialists in peripheral nerve treatment, we have encountered entrapment neuropathy of the upper limb that causes proximal symptoms and named this condition “retrograde neuropathic pain (RNP)” [3]. In daily clinical practice, we also encounter cases consistent with RNP in the lower limbs. An ultrasound-guided SPN block often leads to increases in the dorsiflexion strength of the hallux and aSLR angle. PTN block at the tarsal tunnel can also lead to an increased aSLR angle and improved Bragard test results. These phenomena seem attributable to the effects of RNP and PCS. The relationship between SPN and dorsiflexion strength of the hallux is puzzling given the nerve innervation, but probably related to the adhesion between the peripheral nerve and the muscle or tendon.

We have identified that many patients with low back pain have ankle instability and present three representative cases. Ankle instability was assessed using the anterior drawer test. An ankle sprain is a common injury and develops into CAI in approximately 40-70% of cases [9,10]. A sprain can cause secondary strain on the PTN and SPN [11]. RNP is a known phenomenon in the upper extremities, where distal peripheral neuropathy such as carpal tunnel syndrome causes proximal pain. RNP is a possible explanation for tibial nerve and SPN pathology to cause low back pain.

## Conclusions

We encountered cases of low back pain likely caused by traction neuropathy due to CAI. Because ankle instability is very common, many patients with non-specific low back pain may actually be coming from ankle instability and traction neuropathy. Application of an ankle-foot orthosis and ultrasound-guided nerve block can be useful for both diagnosis and treatment.
